# Oil palm cultivation critically affects sociality in a threatened Malaysian primate

**DOI:** 10.1038/s41598-021-89783-3

**Published:** 2021-05-14

**Authors:** Anna Holzner, Krishna N. Balasubramaniam, Brigitte M. Weiß, Nadine Ruppert, Anja Widdig

**Affiliations:** 1 Department of Human Behaviour, Ecology and Culture, Max Planck Institute for Evolutionary Anthropology, 04103 Leipzig, Germany; 2 Behavioural Ecology Research Group, Institute of Biology, University of Leipzig, 04103 Leipzig, Germany; 3 School of Biological Sciences, Universiti Sains Malaysia, 11800 Pulau Pinang, Malaysia; 4 Department of Population Health and Reproduction, School of Veterinary Medicine, University of California at Davis, Davis, CA 95616 USA; 5 Malaysian Primatological Society, 09000 Kulim, Kedah Malaysia; 6 German Center for Integrative Biodiversity Research, 04103 Leipzig, Germany

**Keywords:** Behavioural ecology, Conservation biology, Ecology

## Abstract

Human-induced habitat alterations globally threaten animal populations, often evoking complex behavioural responses in wildlife. This may be particularly dramatic when negatively affecting social behaviour, which fundamentally determines individual fitness and offspring survival in group-living animals. Here, we provide first evidence for significant behavioural modifications in sociality of southern pig-tailed macaques visiting Malaysian oil palm plantations in search of food despite elevated predation risk. Specifically, we found critical reductions of key positive social interactions but higher rates of aggression in the plantation interior compared to the plantation edge (i.e. plantation areas bordering the forest) and the forest. At the plantation edge, affiliation even increased compared to the forest, while central positions in the macaques' social network structure shifted from high-ranking adult females and immatures to low-ranking individuals. Further, plantations also affected mother–infant relationships, with macaque mothers being more protective in the open plantation environment. We suggest that although primates can temporarily persist in human-altered habitats, their ability to permanently adapt requires the presence of close-by forest and comes with a trade-off in sociality, potentially hampering individual fitness and infant survival. Studies like ours remain critical for understanding species’ adaptability to anthropogenic landscapes, which may ultimately contribute to facilitating their coexistence with humans and preserving biodiversity.

## Introduction

The ongoing expansion of anthropogenic landscapes threatens rainforest ecosystems and the survival of many species^1^. Land conversion for food production and the cultivation of cash crops is the main driver for the global forest loss of estimated 10 million hectares per year^2^. Disturbing natural habitats and presenting sources of anthropogenic food, such modifications create novel and rapidly changing environments for animal populations^3,4^. Habitat fragmentation, hunting and conflicts with farmers are only some of the threats wildlife face in anthropogenic landscape matrices^5,6^. Agricultural lands and urban environments also lack protection through dense forest vegetation and thereby increase exposure to and detection by potential predators^7,8^. With 60% of species being threatened^9^, non-human primates (hereafter ‘primates’) may be particularly susceptible to anthropogenic impact.

Adaptive alterations in behaviour (i.e. behavioural plasticity^10^) are frequently one of the first visible responses of animals to human disturbance. In primates, these responses are diverse and very complex, with most previous studies focusing on their ability to exploit new feeding grounds, shifts in activity or ranging behaviour, and the negative consequences of the human–primate interface, such as increased stress levels among animals or conflicts over resources (reviewed in Ref.^11^). Particularly, plasticity in social behaviour may be critical in determining how well a species can cope in human-altered landscapes, as sociality plays a fundamental role in group-living animals^12^. The majority of primate species, including macaques, live in complex multi-male, multi-female societies, with males leaving their birth group to breed elsewhere, while females are philopatric and form the core of social groups^13^. Strong and enduring social bonds significantly increase fitness in both sexes, with social integration offering energetic benefits and buffering against social and environmental stress^14,15^. Specifically, the quality of affiliative relationships was found to predict individual reproductive performance^16,17^, longevity^18^, and infant survival^19^. One of the most common affiliative interactions among primates, grooming, has a key role in establishing and maintaining social relationships that underlie complex social features such as an animal’s connectedness within the group’s social network^20–22^. Further, juvenile play constitutes a springboard for social competence during the first years of an individual’s life^23^, allowing immatures to construct and expand their social networks, and thereby grow into their social roles as adults^23,24^. On the other hand, agonistic interactions are crucial in social groups, e.g. for the acquisition and maintenance of dominance status which directly impacts individual health^25^. Shifts in any of these behaviours, and (consequently) in individuals’ social network roles, may impair social bonds and thus have downstream effects on group stability and survival^26^.

Behavioural plasticity in the smallest but most essential social units of a group, i.e. mother–infant pairs, may indicate a species’ ability to retain viable populations in anthropogenic environments. Primate mothers provide extensive care to their offspring, and their behaviour strongly affects the development of a wide range of infant behaviours, including environmental exploration, affiliation and aggression, and later sexual and parental behaviour^24,27^. Depending on the social system of a species, but also on individual characteristics such as personality, dominance rank, parity, or infant age and sex^28–31^, mothering styles can vary from highly protective to highly tolerant. Particularly, the reduction of body contact and maternal permissive behaviours are critical components for infant independence^32^. Disruptions of the mother–infant relationship caused by habitat alterations may therefore have severe consequences for offspring health and survival.

Despite their growing significance, quantitative studies on anthropogenic impact on wildlife behaviour often lack comparative assessments across time and space (reviewed in Ref.^33^). A few exceptions have focused on primates that live in (peri-)urban environments, describing substantial variation in activity budgets^34,35^ and individual social behaviour^36–38^ across groups that face varying degrees of human disturbance. In comparison, there is little in-depth knowledge of primate (and indeed wildlife) behaviour in agriculturally modified habitats. Particularly, differences in primate sociality between natural and anthropogenic habitats have not been systematically assessed. Nevertheless, this is crucial to understand in order to assess species’ adaptability to human-modified landscapes and, consequently, to develop effective conservation strategies that will ensure the long-term survival of primates and other species.

Southern pig-tailed macaques (*Macaca nemestrina*, listed as Vulnerable by the IUCN^9^) have lost large parts of their natural forest habitat in Malaysia and Indonesia to oil palm plantations^39^, which today constitute an anthropogenically modified part of their range. Previous studies reported shifts in the macaques’ foraging behaviour when ranging in these monocultures, complementing their diet with palm fruits and actively hunting plantation rats, an excellent source of protein^40,41^. Yet, it remains unclear whether and how the macaques’ sociality in oil palm plantations deviates from their behaviour in the natural, undisturbed forest habitat, and thus potentially impacts their ability to adapt to and survive in this agriculturally modified environment in the long-term. *Macaca nemestrina* has been described as an elusive species that tends to avoid human contact^42^ and may therefore be particularly susceptible to the ongoing clearance of tropical forests.

**Figure 1:**
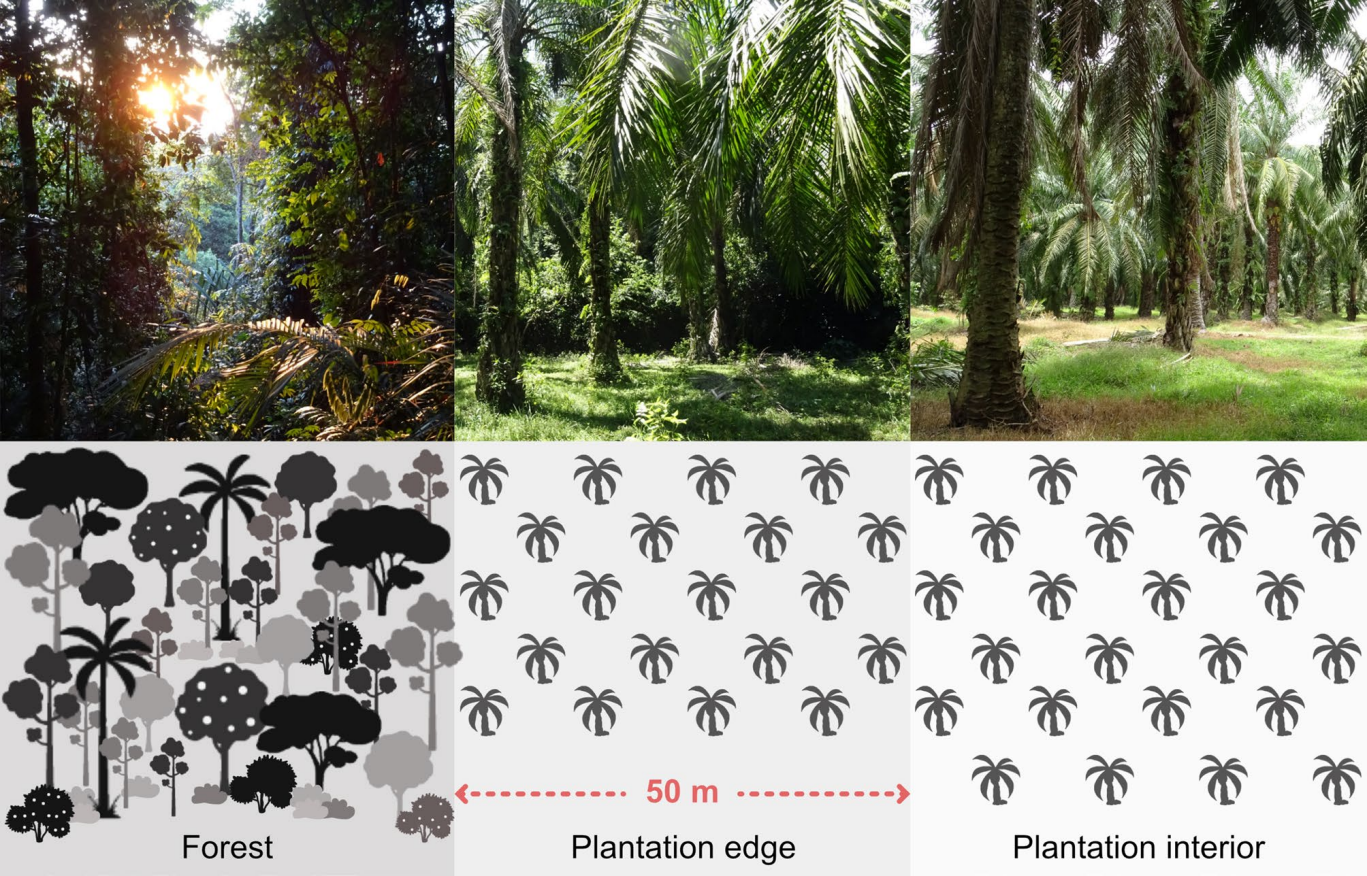
Habitat types at the study site in Segari, Peninsular Malaysia. We distinguished between primary rainforest, the plantation edge, i.e. plantation area within 50 m from the forest border, and the plantation interior, i.e. areas of plantation at a distance of more than 50 m from the forest border. Photos by A. Holzner.

Here, we examined to what extent oil palm monocultures may induce changes in sociality within a population of *M. nemestrina* inhabiting the forest-oil palm matrix of Segari, Peninsular Malaysia. For this purpose, we compared important aspects of the macaques’ social behaviour while ranging in different environments. Whereas previous studies have reported how temporal changes in the overall distribution and abundance of resources may impact aspects of agonistic and affiliative social behaviour within primate groups (e.g. Refs.^43,44^), here we focused on spatial differences in resource availability and their associated changes in sociality. We distinguished between tropical primary forest (i.e. the macaques’ natural habitat), the plantation edge (i.e. oil palm plantation areas within 50 m from the forest border), and the plantation interior (i.e. plantation areas further inside the plantation, Fig. 1). Both plantation habitats provide year-round access to overabundant (i.e. oil palm fruits) and highly valuable (i.e. plantation rats) food sources, and thereby remarkably differ from dipterocarp rainforests, where food is rather scarce and widely scattered^45^. Importantly, the plantation interior poses risks to animals because of lacking shelter, increased visibility to predators and the presence of humans^7,8^, while the plantation edge offers more protection to animals owing to adjacent forest vegetation and the possibility of a close-by, save retreat. Hence, if plantations indeed induce quantitative or qualitative changes in macaque sociality, such changes could be related to shifts in group activity caused by high food availability, and/or effects of the plantation environment itself (e.g. low vegetation cover). In the former case, macaques should show different social patterns in the forest and the plantation, but similar patterns in both plantation environments. In the latter case, sociality in the plantation interior should be different from that observed in the other two habitats.

To quantify how this forest-plantation matrix modifies sociality in *M. nemestrina* and to disentangle potential causes of these changes, our study had four main aims. Firstly, we provide an overview of the macaques’ activity budget in the three different habitats (i.e. forest vs. plantation edge vs. plantation interior), which establishes a premise for investigating and interpreting changes in sociality. Secondly, we assessed habitat-specific differences in rates of affiliative and agonistic interactions within macaque groups. Thirdly, we explored potential changes in the macaques’ social network connectedness when ranging in the plantation habitats compared to the forest. Finally, we compared the mother–infant relationship during the first 6 months after infant birth between habitats, focusing on habitat-induced changes in mothers’ protectiveness.

Based on previous findings^40^, we predicted strong shifts in the macaques’ activity budgets across habitats, with more time spent foraging and feeding in both plantation environments. This may impose time constraints, and consequently induce trade-offs for other behaviours, including socializing^37,38^. Further, we predicted lower rates of affiliative interactions in the plantation interior, which poses greater threats to animals than the protected forest environment and the plantation edge that offers nearby shelter^7,8^. Aggression, on the other hand, was predicted to increase in both plantation habitats as a result of competition over energy-rich food sources (especially plantation rats), yet at a higher rate in the plantation interior based on the assumption that the lack of retreat impedes the avoidance of potential aggressors and likely evokes stress in macaques^34,46^. We further predicted a decrease in the number of individual interaction partners and the connectedness in social networks during visits of the plantation interior. Finally, we predicted macaque mothers to be more protective of their dependent offspring when ranging in the unprotected environment of the plantation interior compared to the forest. Mothers’ protectiveness at the plantation edge, on the other hand, may be intermediate to that observed in the other two habitats.

## Results

### Habitat-specific differences

We studied two habituated groups of macaques inhabiting the Segari Melintang Forest Reserve in Peninsular Malaysia and the surrounding oil palm plantation, with daily plantation visits lasting on average 2.9 hours. Behavioural data were collected in the forest, at the plantation edge and in the plantation interior (see “Methods”).

We measured socio-ecological risks posed by plantation and forest habitat, respectively, by recording flight responses from potential predators shown by the macaques during behavioural data collection. Controlling for observation time, these provide estimates of the probability to encounter predators in the different habitats and thus may serve as a proxy for predation pressure. Similar approaches have been applied in previous studies, linking behavioural responses (e.g. vigilance, response to alarm calls) to risks of predation^47,48^. Accounting for 83% of observed flight behaviour, humans and feral dogs were the most commonly observed threats. Both in the plantation interior and at the plantation edge, flight responses from humans occurred at a rate of approximately 0.1 per hour observation time compared to 0.01 per hour in the forest. Similarly, flight from feral dogs was observed more frequently in the two plantation habitats (0.05 per hour) compared to the forest (0.01 per hour), indicating that macaques are exposed to higher predation risk in the plantation.

**Figure 2:**
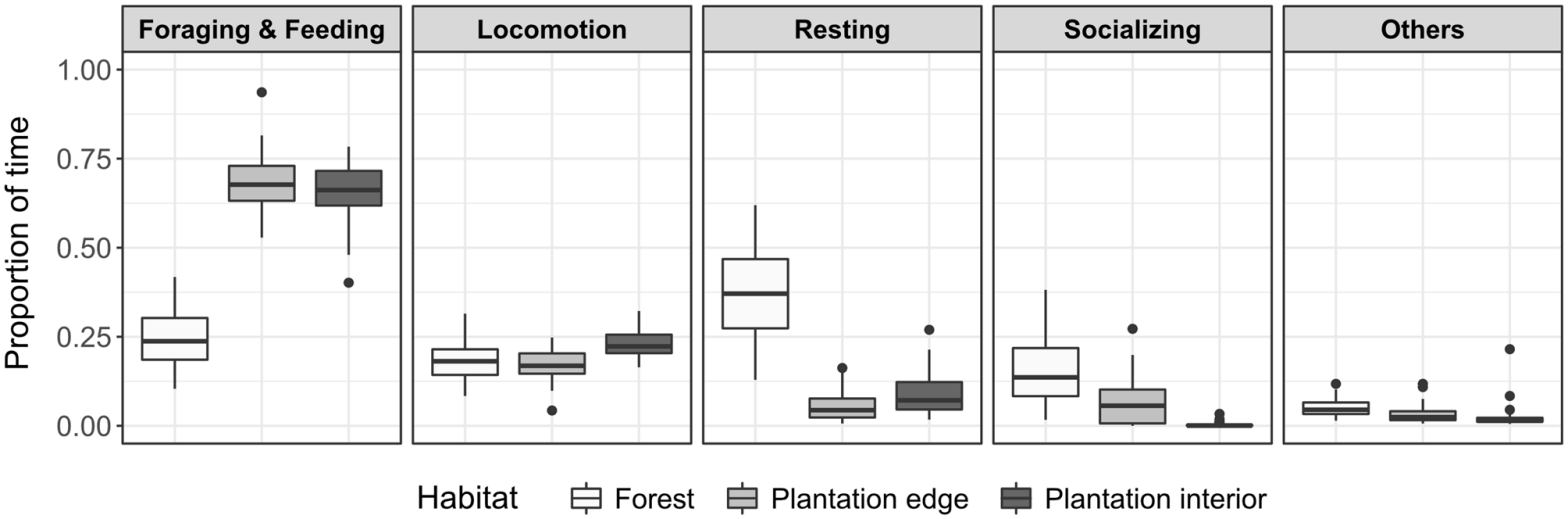
Activity budgets of *Macaca nemestrina* in forest and oil palm plantation. The boxplots indicate the median values and percentiles of individual proportions of time spent for foraging and feeding, locomotion, resting, socializing and other behaviours (e.g. self-directed behaviour, agonistic behaviour, mating), separately for forest, plantation edge and plantation interior. Circles represent outliers. The sample comprised a total of 50 individuals belonging to two social groups.

Overall, we found strong differences in the macaques’ activity budgets across habitats (Fig. 2). All observed activities generally occurred in all three habitats, yet the rates at which they were performed remarkably changed when the macaques were ranging in the oil palm plantation. As predicted, approximately two thirds of the time the macaques spent in the plantation interior and at the plantation edge, respectively, were dedicated to the search and consumption of food (mean ± SD (plantation interior/edge) = 0.65 ± 0.08/0.68 ± 0.08). Hence, the high food availability in oil palm monocultures is equally reflected in a high feeding activity within groups in both plantation habitats.

### Effect of oil palm plantations on macaques’ social interactions

To gain a deeper insight into the effects of human-altered environments on macaques’ social behaviour, we compared rates of affiliative and aggressive interactions between forest and plantation habitats. Among wild primates living in human-modified environments, time constraints imposed by anthropogenic impact, e.g. through increased feeding activity owing to direct human provisioning and/or ranging within agricultural landscapes, may reduce the time available to engage in socializing^36,38^. Further, rates of social interactions may vary across contexts. Aggression, in particular, is known to increase during foraging and feeding which is often associated with high intra-group competition^49^. Therefore, to better assess the direct effect of the plantation environment on primate sociality, we accounted for habitat-specific differences in the macaques’ feeding activity by including individual feeding rates, comprising both foraging and food intake, as a control variable in our statistical models (see “Methods”).

**Figure 3:**
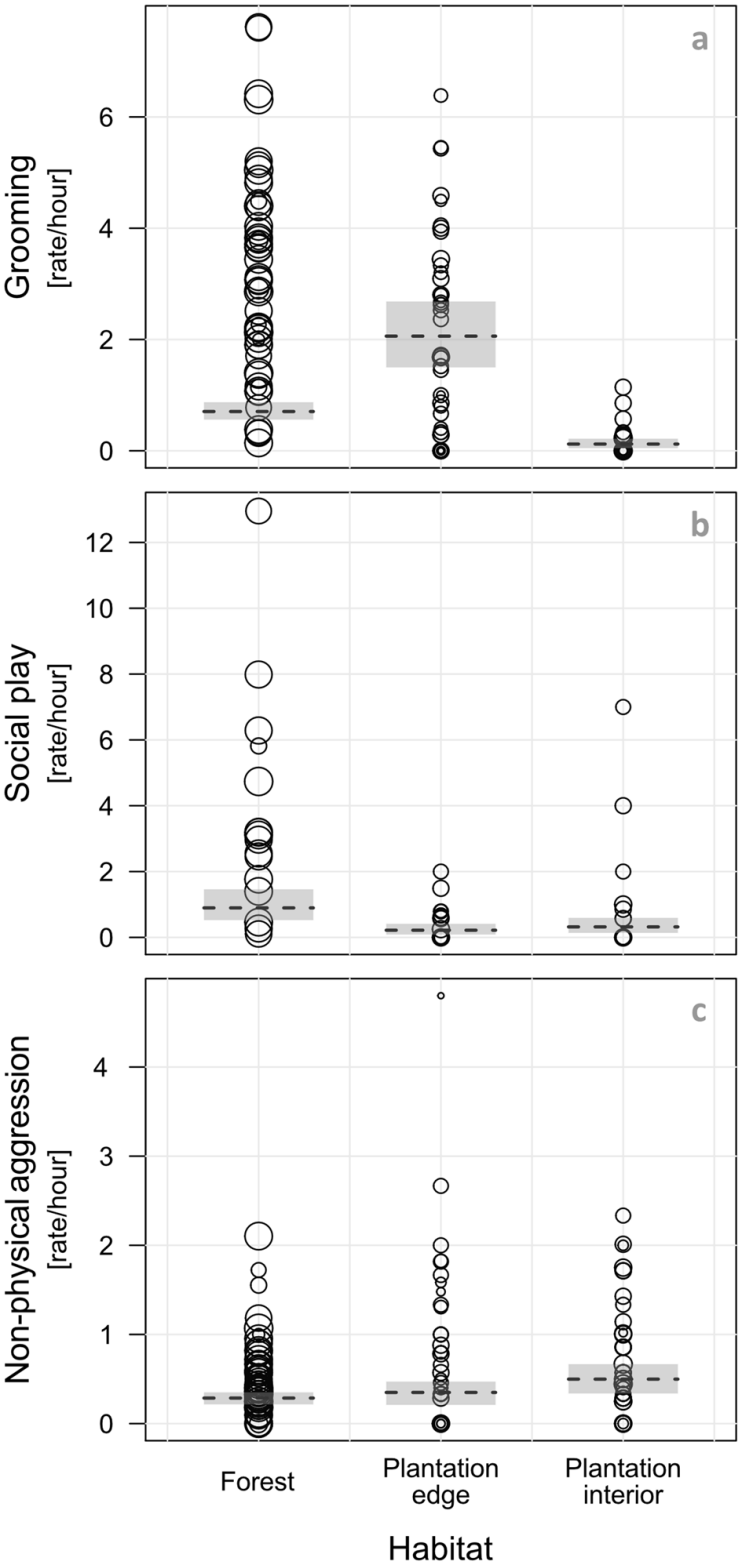
Effect of oil palm plantations on social interactions in *Macaca nemestrina*. Shown are individual rates of grooming (**a**), juvenile social play (**b**) and non-physical aggression (**c**) in the forest, at the plantation edge and in the plantation interior (additional illustration in Supplementary Fig. S1). The dashed lines show the fitted models and the shaded areas their 95% confidence intervals, conditional on continuous control predictors being on their average, and based on age-sex class, group and time of the day manually dummy coded and then centred. For visual clarity, observation values were averaged per individual, with circle areas corresponding to respective observation hours per focal animal (Total N = 1535 focal observations of 50 individuals (36 of *AMY*, 14 of *VOL*) for grooming and non-physical aggression, and 510 focal observations of 16 individuals (14 of *AMY*, 2 of *VOL*) for social play).

Overall, grooming and social play were the most frequently observed positive social interactions, representing 96% of the total time spent socializing. During focal observations, we recorded a total of 1607 grooming bouts and 574 bouts of juvenile social play. The rates of both grooming and social play significantly differed between habitats, while controlling for potentially confounding factors, i.e. the proportion of time spent feeding, an individual’s dominance rank and age-sex class, the study group and time of the day (Likelihood ratio test (LRT, grooming/social play): χ^2^ = 64.48/22.23, df = 2, P < 0.001, N = 1535/510 focal observations of 50/16 individuals, details in Supplementary Table S1). Specifically, grooming rates were critically reduced in the plantation interior, yet significantly increased at the plantation edge compared to the forest (Fig. 3a). Social play, on the other hand, was significantly higher in the forest than in both plantation habitats (Fig. 3b).

As predicted, differences in aggressive behaviour across habitats were generally in contrast to patterns of affiliation, although they were less clear, and varied according to the intensity of aggression. From a total of 496 observed aggressive interactions, 96 included physical aggression, while the others involved more ritualized forms such as facial or vocal threats (hereafter ‘non-physical aggression’). Physical aggression was low in all three habitats and did not significantly differ between forest, plantation edge and plantation interior when controlling for feeding rates, individuals’ dominance rank and age-sex class, the study group and time of the day (LRT: χ^2^ = 3.81, df = 2, P = 0.15, N = 1535 focal observations of 50 individuals). Non-physical aggression was significantly higher in the plantation interior compared to the forest and plantation edge (LRT: χ^2^ = 6.93, df = 2, P = 0.031, N = 1535 focal observations of 50 individuals, Fig. 3c, Supplementary Table S1).

### Effect of oil palm plantations on social network connectedness

In order to capture the impact of habitat alteration on more complex patterns of an individual’s social role that go beyond frequencies of social interactions, we examined differences in the macaques’ social network connectedness when ranging in one of the two plantation environments or the forest. As affiliative interactions were nearly absent in the plantation interior (see Figs. 2, 3a,b), the following analyses focused on describing differences between forest and the plantation edge.

**Figure 4:**
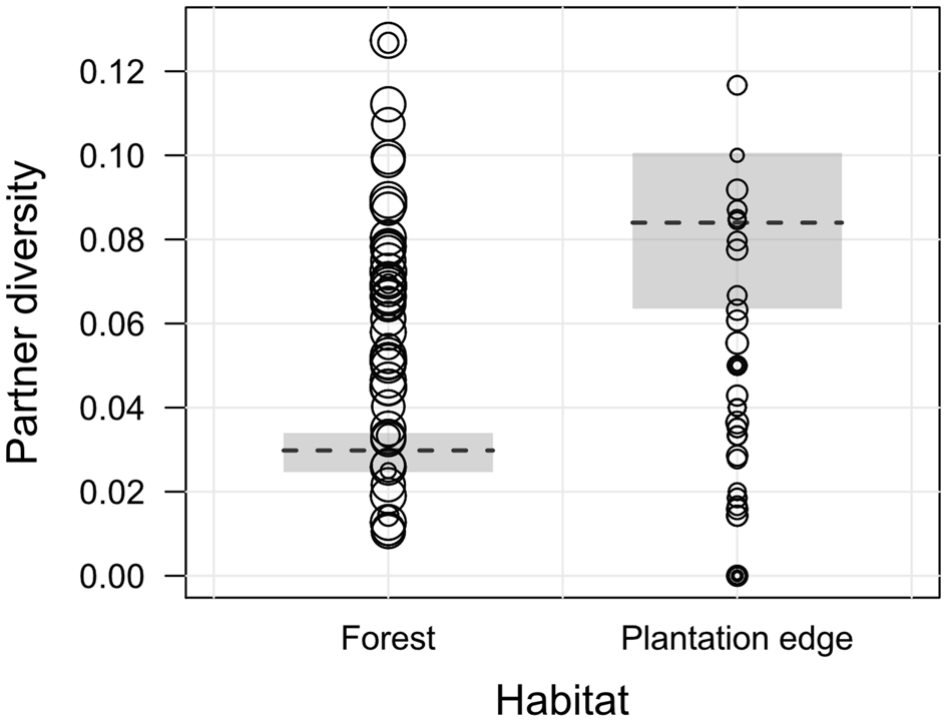
Effect of oil palm plantations on social partner diversity in *Macaca nemestrina*. Shown are individual scores of partner diversity, i.e. the number of different affiliative partners per point time scan, in the forest and at the plantation edge. The dashed lines show the fitted model and the shaded areas its 95% confidence interval, conditional on continuous control predictors being on their average, and based on age-sex class, group and time of the day manually dummy coded and then centred. For visual clarity, observation values were averaged per individual, with circle areas corresponding to respective observation hours per focal animal (Total N = 1206 focal observations of 50 individuals (36 of *AMY*, 14 of *VOL*).

Firstly, we assessed habitat-specific differences in individuals’ social partner diversity, measured as the number of different affiliative social partners per time unit. As described above, we accounted for potential time constraints through increased feeding activity in the oil palm plantation, which may place an overall limit on the time spent engaging in social interactions and hence the number of interaction partners^36^. Partner diversity significantly differed between habitats, when controlling for the respective proportion of time spent feeding, as well as individuals’ dominance rank and age-sex class, the study group and time of the day (χ^2^ = 31.07, df = 1, P < 0.001, N = 1206 focal observations of 50 individuals, details in Supplementary Table S2). Specifically, the number of individuals’ social partners was almost three times higher at the plantation edge compared to the forest (Fig. 4).

**Figure 5:**
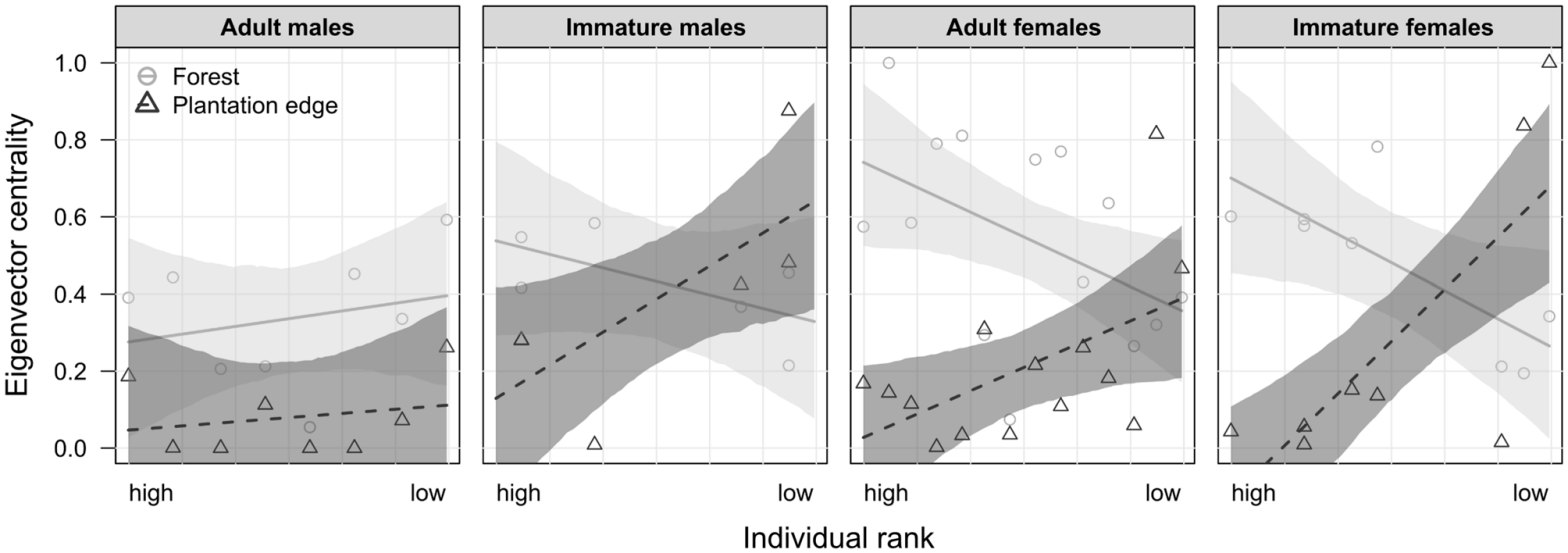
Social network roles of *Macaca nemestrina* in forest and oil palm plantation. Shown are individual scaled scores of eigenvector centrality as a function of individual rank, separately for forest and plantation edge, and for adult and immature males and females, respectively. The lines show the fitted model and the shaded areas its 95% confidence interval (N = 36 individuals).

Secondly, to determine the extent to which an individual’s position in its social group may differ between forest and the plantation edge, we constructed social networks separately for each habitat based on dyadic affiliation, investigating relative changes in individual scores of eigenvector centrality (EC). EC accounts for both direct and indirect network ties, reflecting an individual’s importance in the social network while considering the importance of its neighbours^26,50,51^. It was found to be strongly linked to indicators of individual health and fitness in group-living animals, including macaques^20,51,52^. Therefore, EC serves as a proxy behavioural network measure for determining whether the effects of anthropogenic factors on social behaviour extend beyond simply altering activity budgets, instead potentially impacting their social connectedness (i.e. ties of support), which is directly linked to individuals’ fitness and survival^53,54^. Additionally, we explored whether such differences in network positions might be dependent on an individual’s socio-demographic attributes, particularly its dominance rank and age-sex class. To account for cross-habitat differences in feeding rates (and consequently time available for socializing), we rescaled EC to obtain percentile scores lying between zero (minimum) and one (maximum). As indicated by the statistical model, EC significantly differed between habitats (LRT: χ^2^ = 55.01, df = 8, P < 0.001, N = 36 individuals, details in Supplementary Table S3). The significant three-way interaction between habitat, dominance rank and age-sex class suggests a clear, yet opposite, effect of dominance on EC in different habitats. Specifically, EC decreased with lower dominance in the forest, while it increased with lower dominance at the plantation edge (Fig. 5). In other words, high-ranking individuals were better connected compared to lower ranking individuals when the group was ranging in the forest, while low-ranking individuals occupied the most central positions in the group at the plantation edge. This combined effect of dominance rank and habitat was found to be strongest for immature and adult females, moderate for immature males, and absent in adult males (Fig. 5).

### Effect of oil palm plantations on the mother–infant relationship

Finally, we studied affiliation between macaque mothers and their dependent offspring, as well as maternal behaviour encouraging infant independence during the first 6 months after infant birth. Previous studies on macaques suggested that physical contact between mothers and infants may be dependent upon the mothers’ activity, with foraging and feeding, in particular, but also socializing with conspecifics being associated with decreased contact time^55–57^. In order to ensure that our results are not driven by context-specific variation in maternal behaviour, we included the proportion of time macaque mothers spent in social contact with other group members as a control variable in our statistical models. Controlling for time spent socializing (as opposed to feeding rates) can be expected to make our analysis more conservative given that social contact was observed most frequently in the forest, whereas feeding was the macaques’ main activity in the plantation (Fig. 2).

Overall, our results indicate mothers’ protectiveness to be strongly influenced by the habitat the macaques were ranging in when controlling for respective proportions of time spent socializing, as well as infant age and sex, mothers’ rank, parity and time of the day.

**Figure 6:**
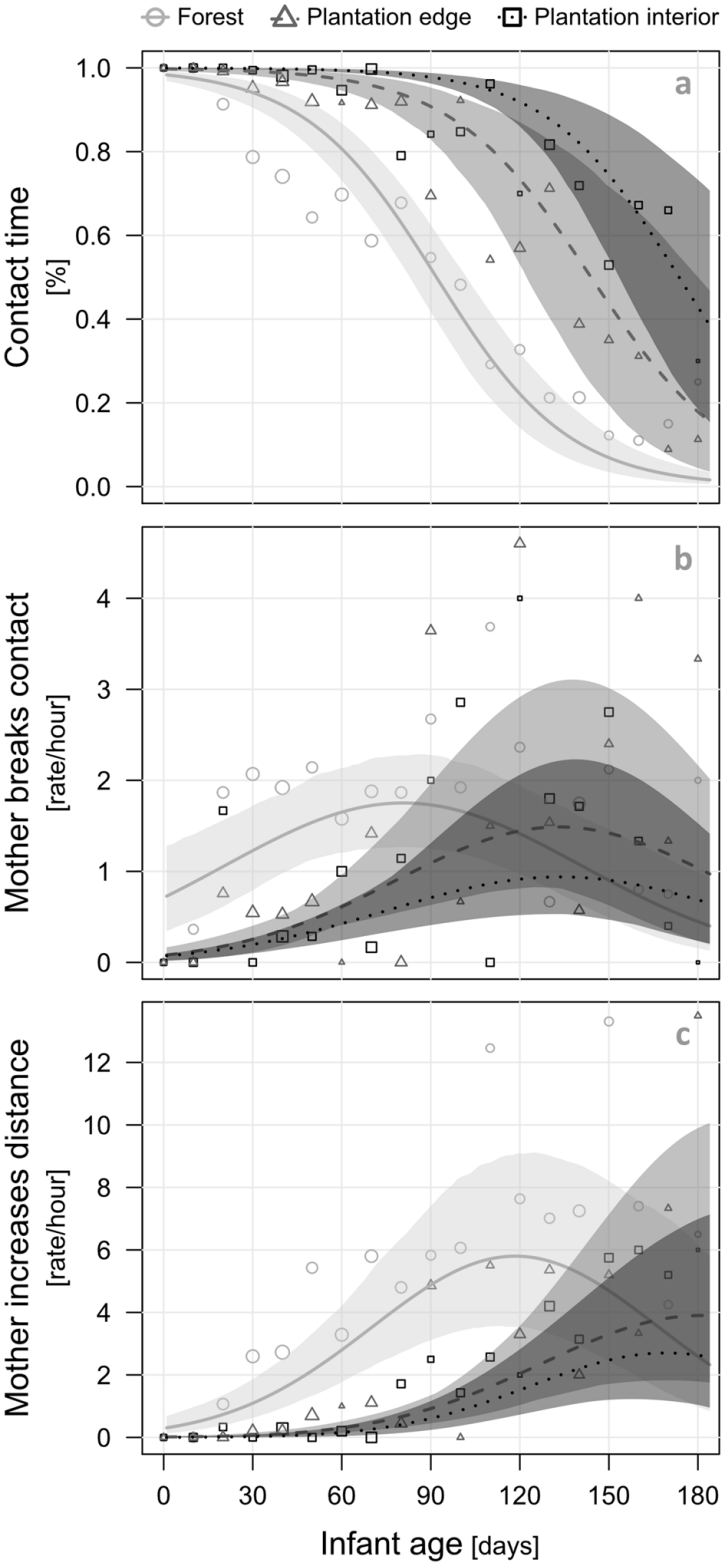
Effect of oil palm plantations on the mother–infant relationship in *Macaca nemestrina*. Shown are the contact time between macaque mothers and their dependent offspring (**a**) and maternal facilitation of infant independence, measured as rates of breaking contact (**b**) and increasing distance (**c**), as a function of infant age, separately for forest, plantation edge and plantation interior. The lines show the fitted models and the shaded areas their 95% confidence intervals, conditional on continuous control predictors being on their average, and based on infant sex, parity and time of the day manually dummy coded and then centred. For visual clarity, infant age was binned into 10-day sections. The area of the points corresponds to the respective sample sizes (Total N = 491 focal observations of 11 mother–infant pairs).

Firstly, the proportion of body contact between macaque mothers and their offspring significantly differed between habitats (LRT: χ^2^ = 46.62, df = 4, P < 0.001, N = 491 observations of 11 mother–infant pairs, details in Supplementary Table S4). As predicted^58^, contact time decreased with infant age, yet the start of this decrease was highly dependent on the habitat (Fig. 6a). Specifically, body contact between mothers and infants already decreased within the first month after infant birth in the forest, after one to two months at the plantation edge, and only after approximately three months in the plantation interior (Fig. 6a).

Secondly, examining the mothers’ incentive to facilitate infant independence, we looked at three different maternal behaviours, i.e. mother breaks contact, mother increases distance, and maternal rejection. With a total of only 15 occurrences during focal observations, rejection was very rare in macaque mothers and thus not considered for multivariate analysis. We further recorded 346 contacts broken by mothers and 838 events of mothers increasing the distance to their offspring. Infant age had a non-linear effect on both maternal behaviours, with the highest rates of breaking contact and increasing distance having been observed at an age between three to five and four to six months, respectively (Fig. 6b,c). As indicated by the full-null model comparisons, the rates of both behaviours were significantly influenced by the habitat (LRT (breaking contact/increasing distance): χ^2^ = 34.72/54.31, df = 6, P < 0.001, N = 491 observations of 11 mother–infant pairs, Supplementary Table S4). Specifically, the significant interaction between habitat and infant age indicates an earlier increase of mothers’ facilitation of infant independence in the forest than in both plantation habitats (Fig. 6b,c).

## Discussion

The present study provides important insights into the effects of anthropogenic environments on primate social behaviour, which is crucial to understand a species’ ability to coexist with humans. Our results demonstrate the presence of critical behavioural alterations in the macaques’ sociality while ranging in the interior of oil palm plantations, compromising on key social interactions, i.e. grooming and juvenile social play, while increasing aggression within groups. In plantation areas close to the forest (i.e. the planation edge), however, both grooming rates and social partner diversity were nearly three times higher than in the forest. Additionally, we found strong habitat-dependent shifts in the macaques’ social network connectedness, with the central positions of high-ranking adult females and immatures of both sexes being passed to low-ranking individuals at the plantation edge. Finally, we found dynamics in the mother–infant relationship, with macaque mothers being more protective in both plantation habitats compared to the forest as indicated by higher proportions of body contact and less maternal behaviour facilitating infant independence.

Generally, observed changes in macaque sociality could also be mediated by habitat-specific differences in food availability rather than being the effect of the plantation environment itself. Therefore, we accounted for variation in the macaques’ feeding activity across habitats by offsetting the time spent feeding during each focal animal sample. Over the long period of data collection (21 months), this is expected to adequately reflect and account for the general amount of time the group engaged in the activity of feeding. Nevertheless, we acknowledge that some variation in the overall activity within groups may remain unaccounted for and consequently, data on group activity would have been a valuable addition to strengthen our results. It is crucial to remind that our study groups represent the first and only non-provisioned population of wild *M. nemestrina* that could be successfully habituated, therefore restricting the observation methods available to answer certain research questions. Within the present study setup, it was not feasible to collect focal and group scan data simultaneously as groups were typically spread out up to 50 m in a dense forest, resulting in low visibility. However (as further detailed below), the distinction between the plantation interior and the plantation edge in fact allowed us to disentangle food-related behavioural changes from direct habitat effects. While food availability and feeding activity were similar in the two plantation habitats, only the planation edge also shared habitat characteristics with the forest (e.g. access to shelter and the opportunity for immediate safe retreat).

Our results confirm previous research, which suggests that oil palm plantations function as foraging and feeding grounds for macaques^40,41,59^. Their motivation to range within these monocultures despite increased predation risk, indicated by rates of flight responses five to ten times higher compared to those observed in the forest, likely lies in the high abundance of food. Previous studies suggested more frequent plantation visits and extended plantation ranges during periods of lower fruit availability in the forest^45,59^. Further, regular plantation visits may be triggered by the high nutritional value of available food sources, as the macaques not only feed on palm fruits but also consume a high number of plantation rats^41^. Yet, this highly valuable food source, coupled with higher visibility and reduced opportunities for lower ranking individuals to hide from or avoid higher ranking potential aggressors, may increase competition and consequently explain higher rates of aggression among macaques in the plantation interior^34,46^. In particular, high-ranking males were frequently observed ‘stealing’ rats from lower ranking males and females of all ranks (A. Holzner, personal observation). Elevated stress levels potentially emanating from anthropogenic environments^38,60^ may further contribute to increasing aggressive interactions. It is also noteworthy that this rise in aggression occurred independently of the increased proportion of time spent feeding in the plantation. Similar rates of aggressive interactions in the forest and at the plantation edge indicate that a high group feeding activity (as found in both plantation habitats) is not necessarily associated with higher levels of aggression. As *M. nemestrina* is hypothesized to occupy an intermediate position between despotic (i.e. less tolerant) and egalitarian (i.e. more tolerant) macaque species^31^, it is not surprising that we found such effects for non-physical threats but not for physical aggression that generally occurred infrequently in our study species.

As predicted, affiliative social behaviour was particularly rare in the plantation interior, i.e. when groups were ranging in plantation areas further than 50 m from the forest border. Specifically, rates of both grooming and juvenile social play were close to zero. Importantly, these results clearly differed from those obtained at the plantation edge. Unlike in the plantation interior, and contrary to our prediction, rates of grooming actually increased at the plantation edge compared to the forest, indicating that a high group feeding activity per se does not prevent individual macaques to engage in affiliative social interactions. Rather, socio-ecological risks posed by plantations (e.g. predation from feral dogs and birds of prey, human contact, or intense competition over highly valuable food sources such as rats^7,8,41^) may discourage the macaques from investing in time-consuming social interactions even when they are not foraging or feeding, in order to be more vigilant against predators and competing conspecifics. Ultimately, this absence of affiliation may have serious implications for animals’ fitness, since both strong social ties as well as a large number of affiliative partners have previously been shown to play a crucial role for individual survival in Cercopithecine primates^61^. Thus, *M. nemestrina*’s ability to persist in mixed oil palm-forest landscapes in the long-term may be strongly constrained if further deforestation forces them into environments where, despite potentially gaining more access to human foods, they lack any possibility to retreat to a nearby forest.

The great value of forest habitat to wild animals is further validated by our findings related to macaques’ behaviour at plantation areas close to the forest. Experiencing shelter through nearby forest vegetation, the macaques not just engaged in social behaviour but actually increased affiliative interactions at the plantation edge compared to the forest. The high investment in grooming, as well as the increased diversity of social partners, may serve as a means to relieve stress that emanates from anthropogenic environments, as highlighted in previous studies^38,62^. In addition, increased affiliation at the plantation edge may heighten levels of social tolerance among group members prior to, or just after visiting the more competitive planation environment.

As previously reported for other wildlife species, including primates, human activity may not only imply shifts in individual rates of social interactions, but further affect more complex social patterns, such as cohesiveness and connectivity in animal social networks^53,54,63^. Recently, Testard et al.^64^ demonstrated that rhesus macaques significantly increased their social network connectedness in the aftermath of a hurricane that caused widespread environmental destruction in their habitat. Here we extend previous findings by revealing how environmental modifications, despite generating a seemingly uniform shift in time spent socializing for entire groups (see above), may *differentially* affect the network connectedness of individual wild animals depending on their socio-demographic characteristics (i.e. their age-sex class and dominance rank), using EC as a measure of social connectedness. In the forest, we observed a gradient in female centrality, with both high-ranking adult females as well as their immature female offspring being the most central, i.e. socially best-connected, individuals. This is consistent with previous studies, reporting top‐ranking females to occupy more central network positions than lower ranking individuals, as they are attractive social partners (e.g. by providing agonistic support in exchange for grooming^65,66^). Remarkably, at the planation edge, this relationship was reversed, possibly because high- and low-ranking females may use different strategies to handle the open, potentially more stressful environment and feeding competition posed by the plantation. Reacting to time constraints, dominants may compromise on their affiliative social connections in order to be more vigilant of competing conspecifics. At the same time, subordinates may increase their affiliative network to gain more tolerance to access energy-rich food sources from dominants. In immature males, we observed a similar, though less strong effect to that observed in females. This is unsurprising, as immature males are still integrated in the maternal network, holding their mothers’ dominance status during childhood, yet they already start to grow into their later role as the dispersing sex^24^. In contrast, EC in adult males was not affected by rank and habitat, which may be closely linked to *M. nemestrina*’s dispersal regime^67^. Whereas philopatric females strongly rely on being well integrated into an intact social network in order to survive and successfully raise offspring^19^, dispersing males have generally more peripheral positions within groups^68^.

Behavioural modifications in the groups’ smallest social units can negatively affect both macaque mothers and their offspring. Ranging in the oil palm plantation daily for an average of almost three hours, the time macaques spend in this human-modified environment corresponds to as much as one fourth of their activity time. With mothers behaving more protectively during plantation visits by increasingly keeping body contact, infants’ independence may be delayed compared to infants growing up exclusively in their natural habitat, i.e. the forest. This could come at an additional cost to mothers, as infant carrying is considered one of the most energetically costly forms of parental care in primates^69^. Further, it is conceivable that inter-birth intervals may become longer, although weaning ages in our study groups (N. Ruppert, unpublished data) did not obviously deviate from the broader literature which suggests that weaning in macaques occurs at about 10–14 months of age^70^. Additionally, alterations in mother–infant bonds may affect the development of offspring in the forest-oil palm matrix. For instance, young males' preparation for their natal dispersal may be hampered by prolonged physical proximity between mothers and infants^30^. Further, increased maternal protectiveness may restrict adolescent females’ opportunities to handle their adult relatives’ infants, and thus to gain and practice maternal skills prior to their first own offspring^71,72^. Ultimately, the disruption of an intact mother–infant relationship through frequent plantation visits may imply negative consequences for offspring health and survival. Long-term data (2014–2018) from our study groups, whose natural habitat have been partly replaced by oil palm plantation already several decades ago, revealed infant mortality within the first year of life to be approximately 55%, with the highest rate (71%) observed in 2016 (N. Ruppert, unpublished data). This is unexpectedly high, considering that infant mortality in other macaque species ranges between 2.7 and 32%^73–78^. However, our data are not sufficient to prove whether infant survival is directly connected to the macaques’ ability to cope with human-induced habitat modifications.

Overall, this study fundamentally contributes to better understanding the impact of oil palm cultivation on sociality in wild primates. We observed behavioural plasticity in the macaques’ overall network structure through to the smallest social units of the group, demonstrating that anthropogenic impacts, even without frequent direct contact with humans, can severely restrict affiliative interactions among macaques and disrupt the mother–infant relationship. These habitat-specific differences in macaque social behaviour cannot be explained by differences in feeding activity alone but are likely to arise from the lack of protection through a nearby refuge, the forest. Decreased individual fitness and high rates of infant mortality may ultimately cause difficulties for threatened species to maintain their viable population size. In this context, it is essential to protect the remaining populations and facilitate their coexistence with humans in anthropogenic landscapes. As umbrella species, primates represent a wide range of wildlife that depends on primary rainforest^79^. Hence, their protection will ultimately contribute to maintaining biodiversity and important ecosystem functions of tropical habitats. Unravelling the effects of both direct and indirect anthropogenic disturbances on primate social behaviour can serve as a basis for understanding the degree to which a species can adapt to human-altered habitats and may aid in developing effective conservation strategies and species management plans. Looking at the most important affiliative behaviours in primates, our results suggest that proximity to the forest is the key factor for macaques to be able to perform the full range of their natural behavioural repertoire. Maintaining forest corridors, an important conservation tool to create viable interfaces between forests and agricultural landscapes, may therefore not only facilitate natural dispersal and link fragmented wildlife populations affected by monocultures, but also enable animals to engage in essential social behaviours that directly affect their fitness. Ultimately, this will contribute to improving individual well-being and ensuring the long-term survival of primates and other species.

## Methods

### Study site and subjects

From January 2017 to September 2018, we collected non-invasive observational data on two habituated groups of wild southern pig-tailed macaques (*Macaca nemestrina*) at the Segari Melintang Forest Reserve (SMFR) and the oil palm plantations bordering its south-western edge (4° 19-20′ N, 100° 34-36′ E). The size and composition of the groups slightly changed during the study period, either due to male immigration or dispersal^80^, animals dying or being born, or juveniles reaching sexual maturity. During the study period, group 1 (named *AMY*) consisted of 5–8 adult males, 12–15 adult females and 18–23 immature individuals. Group 2 (named *VOL*) consisted of 11–14 adult males, 19–21 adult females and 16–18 immature individuals. The age and sex of individual macaques were known from long-term observations^40,41^. Both groups visited the plantation area bordering their forest habitat almost daily (mean ± SD (*AMY/VOL*) = 3.1 ± 1.8/2.7 ± 1.8 h per day^41^). The annual home ranges of group *AMY* and *VOL* were 92.7 and 96.6 hectares, respectively, with used plantation areas accounting for approximately one third of the total home range areas^41^. As group *VOL* has not been fully habituated before the start of 2018, assessments of the macaques’ social network and the mother–infant relationship were performed only on group *AMY*.

To date, our study groups represent the only wild population of this species which could be successfully habituated, probably relating to the generally shy and elusive nature of *M. nemestrina*^42^. Nevertheless, the present setup of comparing the same groups of macaques across different habitats may be advantageous over an approach investigating cross-group differences, by ruling out the effects of group-specific factors, such as variation in group size, sex ratio, or cross-population genetic differences.

### Habitat types

In order to assess the impact of anthropogenic environments on the macaques’ social activities, we divided the home range areas of our study groups into three habitats, i.e. forest, plantation edge and plantation interior (Fig. 1). SMFR comprises 2742 hectares of which 408 hectares are strictly protected Virgin Jungle Reserve. Its main vegetation types are dipterocarp lowland forest and alluvial fresh-water swamp^40^. The 420-hectare sized oil palm plantation bordering the reserve was established between 1980 and 1990 and is managed by a federal authority. The oil palm estate was accessible to macaques, with encounters between wildlife and plantation workers being occasionally observed, yet these did not involve regular conflicts such as hunting or chasing. We defined ‘plantation edge’ as the plantation area which is located within 50 m from the forest border, whereas ‘plantation interior’ refers to all plantation areas further than 50 m from the forest border. This distinction was made to account for the fact that plantation areas in close proximity to the forest provide additional shelter and protection for the macaques through close-by forest vegetation. So-called ecotones that form transitional areas between two distinct ecological habitats were reported to be of great environmental importance, potentially serving as speciation and biodiversity centres^81^. We chose the distance of 50 m according to the average diameter of the macaque groups’ dispersion (edge-centre-edge).

### Behavioural data collection

We collected data using 30-min focal animal sampling^82^ based on a species-specific ethogram established for the study species (adapted from Ref.^83^) in the forest, at the plantation edge and in the plantation interior. We observed a total of 50 individually recognizable macaques (36 of group *AMY*, 14 of group *VOL*). Focal individuals were chosen to represent all age-sex classes. The order of focal observations was randomized, aiming at sampling each individual only once per day. If a focal animal entered another habitat during a 30-min sampling protocol or went out of sight for more than 10 min, this observation was stopped. Incomplete protocols were considered for multivariate analysis if they lasted at least 15 min. Total observation time was 724 h (mean ± SD = 14.5 ± 3.6 h per subject).

To assess socio-ecological risks posed by forest and plantation habitats, we recorded the occurrence and duration of flight behaviour shown by the macaques in response to the presence of potential predators, i.e. humans, feral dogs, birds of prey, snakes or monitor lizards, as a proxy for the probability to encounter these predators. To provide an overview on shifts in the macaques’ overall activity budget, we continuously recorded their activity in the different habitats. Recorded activities included feeding (i.e. ingesting food), foraging (i.e. searching for or manipulating food), locomotion (i.e. walking, running, climbing or travelling without other activity), resting (i.e. standing, sitting or lying without other activity, eyes may be open or closed), socializing (i.e. all positive social interactions, e.g. grooming, being groomed and groom presenting, social play and huddling) and others (e.g. sexual and agonistic interactions or self-grooming). To assess individual differences in affiliation, we recorded the rate, measured as number per focal observation, and duration of all bouts of grooming and social play between the focal subject and other group members. As measures of rates and durations were highly correlated (Pearson’s r (grooming/social play) = 0.78/0.81, P < 0.001), we considered only rates for analyses. Further, we recorded aggressive behaviour exchanged between the focal subject and other group members, considering both physical (i.e. attack, bite, grab, hit, push) and non-physical aggression (i.e. charge, chase, lunge, stare and vocal or open mouth threat). Social data were complemented by ad libitum data^82^ on aggression, displacement and submission among adult males and adult females for the purpose of constructing dominance hierarchies (see below). Data on social interactions included information on both the initiator and the recipient. Following previous studies^84^, a repetition of a behaviour was scored as a new bout if more than ten seconds had elapsed between occurrences or at least one partner had switched to a mutually exclusive activity (e.g., from grooming to aggression). During an aggressive event in which a number of different agonistic patterns occurred in quick succession, only the most intense kind of aggression was considered for analyses^84^.

To assess social partner diversity and affiliative social networks across different habitats, we recorded data on spatial proximity between macaques. We took point time scans^82^ every 3 min within the 30-min sampling protocol, recording all group members in body contact with the focal individual. We further recorded whether or not this contact resulted from an affiliative interaction (e.g. during grooming, play or huddling). This was the case for 98.3% of our observations. The total number of scans recorded was 14,205 (mean ± SD = 284 ± 71 scans per subject).

To assess the mother–infant relationship, we additionally observed eleven mother–infant pairs from group *AMY* in the three different habitats for the first six months after infant birth. Total observation time was 240 h (mean ± SD = 21.8 ± 9.4 h per mother–infant pair). We continuously recorded maternal behaviour promoting infant independence^58^. Specifically, we recorded the number of contacts broken (i.e. any movements disrupting body contact between mother and infant), increases of distance (i.e. movements increasing the distance between mother and infant from within arm’s reach (about 60 cm) to outside of arm’s reach) and maternal rejection (i.e. attempts by the infant to make contact that were rejected by the mother, e.g. by turning, running away, or holding the infant at a distance with an arm^58^). To ensure independence between these measures, increases of distance were only recorded if at least 5 s elapsed since contacts were broken. To assess spatial proximity in mother–infant pairs, we took point time scans^82^ every minute within the 30-min sampling protocol, recording whether or not mothers and their infants stayed in body contact, including ventro-ventral contact, nipple holding, side-by-side contact, and grooming. In total, we recorded 14,365 scans (mean ± SD = 1306 ± 561 scans per mother–infant pair). Further, we assessed variation in maternal rates of socializing with conspecifics across habitats, recording every three minutes during focal observations whether or not mothers were involved in an affiliative interaction (e.g. grooming or huddling).

### Location data

We collected location data with a Garmin GPSMAP62s daily from January to December 2017 for group *AMY* and from October 2017 to September 2018 for group *VOL*. We took GPS waypoints every minute while following the macaques. During behavioural observations, we further took the coordinates of each focal observation at half-time of the respective focal protocol. Annual home range areas of group *AMY* and *VOL* were created following Holzner et al.^41^, by using point Kernel Density Estimations (KDE) with 95% probability of use^85^. Home range analyses were conducted with the Home Range Analysis and Estimation (HoRAE^86^) toolbox of the GIS software OpenJUMP (version 1.14^87^). To provide an overview on the occurrence of affiliative and aggressive social interactions across different habitats within the macaques’ home ranges, we created interpolation maps (see Supplementary Fig. S1) based on mean behavioural rates occurring during focal observations per 50 m × 50 m grid cell using the Inverse Distance Weighting (IDW) tool of the software QGIS (version 3.12^88^).

### Dominance hierarchy

From 948 dyadic agonistic interactions with a clear winner and loser outcome collected during focal and ad libitum observations, we estimated rank orders using David’s scores^89^. These were obtained in R (version 3.4.4^90^) using the function *DS* from the package ‘EloRating’ (version 0.46.8^91^). We set argument ‘prop’ to ‘Dij’, calculating dyadic win proportions corrected for chance^92^. As in macaques rank acquisition and function typically differ between sexes, with non-natal males fighting for dominance, while females socially inherit the rank of their mothers^93^, we estimated rank orders separately for males and females. Following Kaburu et al.^94^, we controlled for differences in group size and sex ratio by standardizing dominance rank as ‘(Rank-1)/(N − 1)’, where N represents the number of focal animals per group and sex class. Standardized dominance rank values range from zero (top-ranking individual) to one (bottom-ranking individual). Referring to literature^95^, immature males and females got assigned the same rank as their biological mother, or, if their mother already died, the rank of their closest adult female relative.

As David’s scores do not account for potential temporal fluctuations in rank orders, we further assessed rank stability using the function *stab.elo* of the package ‘EloRating’ (version 0.46.8^91^). Indices range between zero (unstable) and one (stable), reflecting to what extent rank positions of individuals change over time. Males and females of both groups displayed stability indices close to one (males (*AMY*) = 0.9950, females (*AMY*) = 0.9956, males (*VOL*) = 0.9909, females (*VOL*) = 0.9943), suggesting highly stable dominance hierarchies during the sampling period in both sexes. Therefore, David’s scores seem to be appropriate for estimating dominance ranks in our study groups.

### Social network analysis

Based on affiliative interactions observed during individual focal sampling, we constructed the social network of group *AMY* separately for forest and plantation habitats. Following Lehmann et al.^26^, we assessed dyadic affiliation as the proportion of scans two individuals were in social contact (i.e. grooming, social play or affiliative body contact). We created social networks in R (version 3.4.4^90^) using an undirected data structure with the function *graph_from_data_frame* from the package ‘igraph’ (version 1.2.5^96^). For each individual, we extracted the eigenvector centrality (EC), a commonly used network parameter to quantify individual social connectedness^26,50^. EC is a measure of both direct and indirect network ties, reflecting a node’s importance while considering the importance of its neighbours. Thus, a high value of EC suggests that an individual has many social partners who themselves also have many partners. As we were particularly interested in an individual’s connectedness in relation to other group members, we rescaled the obtained values of EC in each habitat to get percentile scores lying between zero (minimum) and one (maximum), as opposed to investigating absolute scores.

### Statistical analysis

Multivariate statistical analyses assessing the impact of different habitats on rates of affiliation and aggression (models 1–4), social partner diversity (model 5) and network connectedness (models 6), and the mother–infant relationship (models 7–9) were conducted in R (version 3.4.4^90^) using Generalized Linear Mixed Models (GLMM^97^). Models were fitted using the functions *lmer* and *glmer* of the package ‘lme4’ (version 1.1.19^98^) with the optimizer *bobyqa.* To facilitate model interpretation and convergence, we standardized all continuous predictors before model fitting to a mean of zero and a standard deviation of one^99^. Full-null model comparisons were derived using likelihood ratio tests (LRT^100^) using the R function *anova* with argument ‘test’ set to ‘Chisq’. Tests of individual fixed effects were derived using the R function *drop1* with argument ‘test’ set to ‘Chisq’, yet control predictors were not interpreted. Confidence intervals were derived using the function *bootMer* of the package ‘lme4’ (version 1.1.19^98^), using 1000 parametric bootstraps.

Rates of affiliation and aggression (models 1–4): To investigate the impact of the habitat on the display of grooming, juvenile social play, and physical and non-physical aggression, we constructed four GLMMs^97^ with Poisson error structure and log link function. As response variables we used the number of grooming bouts (model 1), bouts of juvenile social play (model 2), bouts of physical aggression (model 3), and bouts of non-physical aggression (model 4) per focal observation (N = 1535 focal observations of 50 individuals for models 1, 3 and 4, and N = 510 focal observations of 16 immature individuals for model 2). The models included the habitat (forest, plantation edge or plantation interior) as a fixed effect test predictor, while controlling for the proportion of time spent feeding during each focal protocol. In doing so, we accounted for potential time constraints imposed by increased feeding activity in agriculturally modified environments^36,38^, as well as for context-specific variation in the probability to engage in social interactions^49^. Further, we controlled for individual dominance rank and age-sex class (adult male, adult female, immature male or immature female), as both rank and age-sex class have previously been shown to affect the occurrence of affiliative and agonistic interactions in macaques^24,65,101^. To account for changes in the overall group activity throughout the day, inter-group variation and repeated observations of the same individuals, we further included the time of the day, divided into four time blocks (early morning: 7:00–09:59 a.m., late morning: 10:00 a.m.–12:59 p.m., early afternoon: 13:00–15:59 p.m. or late afternoon: 16:00–18:59 p.m.) and macaque group (*AMY* or *VOL*) as fixed effect control predictors, and the focal individual ID and sampling date as random effects. Additionally, we included the random slopes of habitat and time of the day within focal individual in models 1–4, and the random slope of rank within sampling date in models 1, 3 and 4^100,102^. Controlling for differences in the sampling effort, we further included the duration of each focal observation as an offset term into the models^103^. To test the effect of different habitats, we compared the full models with respective reduced models lacking only our test predictor (habitat) using a LRT^100^.

Social partner diversity (model 5): To investigate the impact of the habitat on individuals’ social partner diversity, defined as the number of affiliative social partners per time unit, we constructed a GLMM^97^ with Poisson error structure and log link function. In order to reduce interdependencies of partner diversity measures, we determined the number of partners at the level of focal protocols (rather than extracting the degree of each individual at the network level)^38^. Accordingly, we used the number of different social partners per focal observation as response variable (N = 1206 observations of 50 individuals). We included the habitat (forest or plantation edge) as a fixed effect test predictor and, consistent with models 1 and 2, the proportion of time spent feeding, individual dominance rank and age-sex class, the time of the day, and macaque group as fixed effect control predictors. We further included the focal individual ID and sampling date as random effects, while considering the random slopes of habitat and time of the day within focal individual and the random slope of rank within sampling date^100,102^. Controlling for differences in the sampling effort, we included the number of point time scans per focal observation as an offset term into the model^103^. To test the effect of different habitats, we compared the full model with a reduced model lacking only our test predictor (habitat) using a LRT^100^.

Social network connectedness (model 6): To investigate the impact of the habitat on a common network parameter, i.e. the EC defined above, we constructed another GLMM^97^ with Gaussian error structure. As response variable we used the individuals’ scaled EC (model 6) in each habitat (N = 68 observations of 34 individuals). We included the habitat (forest or plantation edge) and its interactions with individual dominance rank and age-sex class (as defined above) as fixed effects, and the focal individual ID as random effect. To test the effect of different habitats on EC, we performed a LRT^100^, comparing the full model with a reduced model lacking our test predictor (habitat) and its interactions with dominance rank and age-sex class.

To account for interdependency of the network measure (i.e. EC) used as outcome variable in our GLMM, we used a node-swapping permutation approach, based on 1000 permutations of the outcome variable^104^. This included recalculating the network parameter after randomly swapping the nodes of the original networks. We used node-swapping (as opposed to generating random graphs or using pre-network ‘edge-swapping’ randomizations) since this approach seemed better suited for our purposes of testing regression-based null hypotheses in a taxon with a largely stable group composition and relatively low observation biases^105,106^. Specifically, node-swapping preserves the overall size, number of connections, and structure of the network, thereby also preserving the overall distribution of node-level measures such as EC. It is therefore a more conservative approach that may be less susceptible to Type I errors, compared to random graph generation or edge-swapping^105,106^. After each permuted swapping event, we refit the same GLMM using the newly created parameter as response variable. Comparing the observed model coefficients with the distribution of the 1000 permuted coefficients, we calculated p-values as the number of times the coefficient value of the observed network is smaller than a randomized network, divided by the number of randomizations^104^.

Mother–infant relationship (models 7–9): To investigate the impact of the habitat on the mother–infant relationship, we constructed three GLMMs^97^, with the proportion of body contact between mothers and offspring (model 7), the number of maternal breaks of contact (model 8), and the number of maternal increases of distance (model 9) per focal observation being the response variables (N = 491 focal observations of 11 mother–infant pairs for models 7–9). Investigating effects on the proportion of time spent in contact (model 7), we used a GLMM^97^ with binomial error structure and logit link function. In R, this analysis of proportions is possible by using a two-columns matrix with the number of contacts and non-contacts per individual as the response^97^. Models 8 and 9 were created using a count response with Poisson error structure and log link function. Here, we controlled for differences in the sampling effort by including the duration of each focal observation as an offset term^103^. In all three models, we included the habitat (forest, plantation edge or plantation interior) as a fixed effect test predictor, while controlling for the proportion of time mothers spent socializing with conspecifics during each focal protocol in order to account for context-specific variation in maternal behaviour^56,57^. Further, we controlled for infant and maternal characteristics, which were previously shown to affect the mother–infant bond, i.e. infant age^58^ and sex (male or female^30^), as well as maternal rank and parity (primiparous or multiparous^28,29^). As in models 1–5, we accounted for changes in the overall group activity over the day by including the time of the day as a fixed effect control predictor. Further, we included the mother–infant pair and sampling date as well as the combination of these two as random effects, as mother–infant pairs were frequently observed more than once on a given day. Additionally, we included the random slopes of habitat, infant age and time of the day within the mother–infant pair^100,102^. As we expected infant age to have a non-linear effect on the rates of maternal breaking contact and increasing distance, we additionally incorporated squared infant age into models 8 and 9. Further, we included the two-way interaction between habitat and infant age in model 7, and its interactions with infant age and squared infant age in models 8 and 9. To test the effect of different habitats, we compared the full models with the respective reduced models lacking our test predictor (habitat) and its interactions with infant age and squared infant age, respectively, using LRTs^100^. In case of a non-significant interaction, we re-ran the model without the interaction term to facilitate the interpretation of the main effects in the model.

For models 1–9, we assessed model stability by excluding the levels of the random effects one at a time and comparing the estimates from the obtained models with the estimates from the model based on all data using a self-written R function provided by Roger Mundry. This did not indicate any obviously influential case. To rule out collinearity, we determined Variance Inflation Factors (VIFs) for respective standard linear models excluding the random effects using the function *vif* of the package ‘car’ (version 3.0.2^107^). According to these, none of the models indicated issues regarding collinearity (all VIFs < 1.50). We confirmed that overdispersion was no issue in all models with a Poisson (models 1–4, 5, 8, 9) or binomial (model 7) error structure. Yet, there was an indication of underdispersion in model 3, probably reflecting the low rates of physical aggression in our dataset. Further, there was indication of slight underdispersion in models 7–9, likely due to limited data points (dispersion parameters = 0.89/0.93/0.38/0.73 for models 1–4, 0.89 for model 5 and 0.51/ 0.68/ 0.54 for models 7–9). Visual inspections of a QQ-plot^108^ of the residuals and a scatterplot of the residuals plotted against the fitted values^109^ did not reveal obvious deviations from the assumptions of normally distributed and homogeneous residuals in the Gaussian model (model 6).

### Ethical note

We obtained permits to study *Macaca nemestrina* from the Department of Wildlife and National Parks Peninsular Malaysia (permit holder: Dean of School of Biological Sciences, Universiti Sains Malaysia). We obtained permits to enter the forest reserve bordering the oil palm plantation from the Forestry Department Peninsular Malaysia (permit holder: Asyraf Mansor, School of Biological Sciences, Universiti Sains Malaysia). No written permit was needed to enter the plantations, but we informed the local management about the study. This non-invasive study was conducted in line with Universiti Sains Malaysia’s animal ethics requirements.

## Supplementary Information

Supplementary Information.

## Data Availability

The datasets generated and analysed during this study are available on Zenodo: 10.5281/zenodo.4740726.
