# Supplementary material for: Oil palm cultivation critically affects sociality in a threatened Malaysian primate
Source: Sci Rep. 2021 May 14;11:10353. doi: 10.1038/s41598-021-89783-3 (PMC8121792; doi:10.1038/s41598-021-89783-3)
Supplement: Supplementary file 1 — Supplementary Information. [file 41598_2021_89783_MOESM1_ESM.pdf]

Supplementary Information

Oil palm cultivation critically affects sociality in a threatened Malaysian primate

Anna Holzner, Krishna N. Balasubramaniam, Brigitte M. Weiß, Nadine Ruppert, Anja Widdig

Supplementary Figure

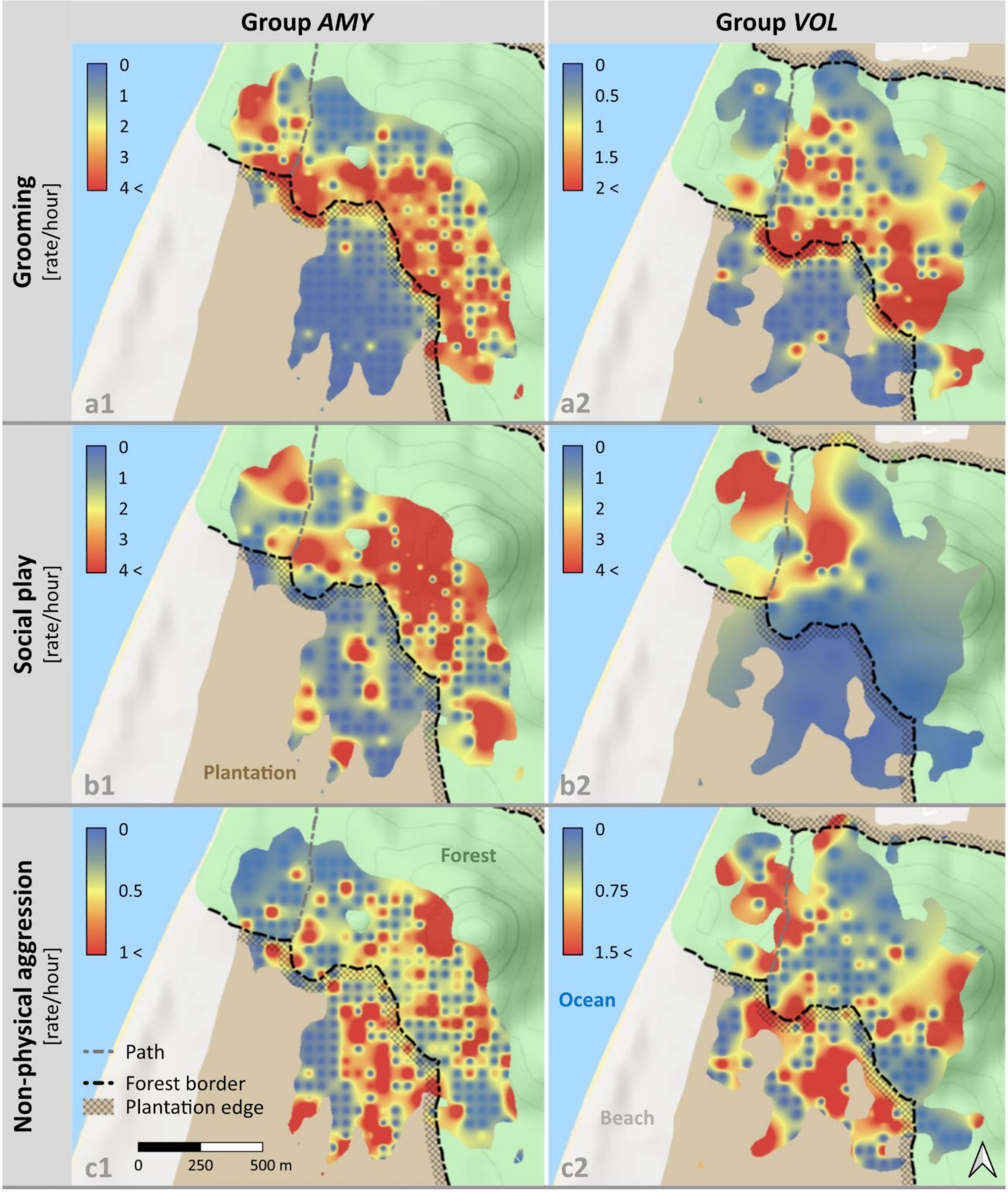

Figure S1. Affiliative and aggressive interactions among southern pig-tailed macaques in forest and oil palm planation. Interpolation maps indicate the mean rates per hour of grooming (a), juvenile social play (b) and non-physical aggression (c) occurring during focal observations per 50 m x 50 m grid cell within the home range areas of group *AMY* (1) and group *VOL* (2). The sample comprised a total of 1,535 focal observations of 50 individuals (36 of *AMY*, 14 of *VOL*) for grooming and non-physical aggression and 510 focal observations of 16 individuals (14 of *AMY*, 2 of *VOL*) for social play. Home range areas were established using the Home Range Analysis and Estimation (HoRAE) toolbox of the GIS software OpenJUMP (version 1.14, http://www.openjump.org). Interpolation maps were created using the Inverse Distance Weighting (IDW) tool of the software QGIS (version 3.12, http://qgis.osgeo.org).

Supplementary Tables

Table S1. Results of the GLMMs exploring the effect of the habitat on the rates of grooming (model 1), social play (model 2) and non-physical aggression (model 4) in *Macaca nemestrina*. Shown are model estimates, standard errors (SE), lower and upper confidence intervals (CI) and test results of individual effects. Control variables are not interpreted. Significant p-values are shown in bold. As the full-null model comparison of model 3 (physical aggression) did not reveal significance (LRT habitat: χ^2 = 3.81, df = 2, p = 0.15), no details about individual effects are presented.

Predictor variable	Estimate	SE	lower CI	upper CI	X^2	P
Grooming (model 1)						
Intercept	-1.30	0.23	-1.76	-0.85		
Habitat (forest vs. plantation edge)[a]	1.07	0.16	0.72	1.39	64.48	**< 0.001**
Habitat (forest vs. plantation interior)[a]	-1.76	0.38	-2.65	-1.17		
Control variables						
Feeding rate[b]	-1.29	0.07	-1.43	-1.15	121.31	**< 0.001**
Age-sex class (adult ♂ vs. adult ♀)[c]	1.25	0.22	0.81	1.69		
Age-sex class (adult ♂ vs. immature ♂)[c]	1.05	0.29	0.47	1.66	30.24	**< 0.001**
Age-sex class (adult ♂ vs. immature ♀)[c]	1.48	0.27	0.99	2.00		
Rank[d]	0.03	0.09	-0.16	0.20	0.08	0.78
Group (*AMY*=0, *VOL*=1)	-0.66	0.21	-1.09	-0.25	9.09	**0.003**
Daytime (early morning vs. late morning)[e]	0.07	0.11	-0.14	0.29		
Daytime (early morning vs. early afternoon)[e]	0.09	0.13	-0.15	0.35	8.72	**0.033**
Daytime (early morning vs. late afternoon)[e]	0.45	0.14	0.16	0.73		
Social play (model 2)						
Intercept	1.14	0.37	0.39	1.84		
Habitat (forest vs. plantation edge)[a]	-1.41	0.32	-2.19	-0.86	22.23	**< 0.001**
Habitat (forest vs. plantation interior)[a]	-1.03	0.31	-1.70	-0.48		
Control variables						
Feeding rate[f]	-0.46	0.14	-0.72	-0.18	7.94	**0.005**
Age-sex class (♂ = 0, ♀ = 1)	-1.18	0.41	-1.92	-0.33	7.14	**0.008**
Rank[g]	-0.10	0.20	-0.50	0.29	0.24	0.63
Group (*AMY*=0, *VOL*=1)	-1.35	0.77	-3.34	0.02	2.96	0.085
Daytime (early morning vs. late morning)[e]	-0.60	0.31	-1.18	-0.04		
Daytime (early morning vs. early afternoon)[e]	-0.60	0.32	-1.22	0.03	6.61	0.085
Daytime (early morning vs. late afternoon)[e]	-0.36	0.25	-0.86	0.14		
Non-physical aggression (model 4)						
Intercept	-0.71	0.22	-1.18	-0.31		
Habitat (forest vs. plantation edge)[a]	0.20	0.22	-0.28	0.60	6.93	**0.031**
Habitat (forest vs. plantation interior)[a]	0.55	0.20	0.15	0.93		
Control variables						
Feeding rate[b]	-0.09	0.09	-0.27	0.09	0.87	0.35
Age-sex class (adult ♂ vs. adult ♀)[c]	-0.67	0.16	-1.00	-0.34		
Age-sex class (adult ♂ vs. immature ♂)[c]	-0.23	0.22	-0.71	0.20	23.18	**< 0.001**
Age-sex class (adult ♂ vs. immature ♀)[c]	-0.95	0.22	-1.43	-0.48		
Rank[d]	-0.16	0.07	-0.30	-0.03	4.58	**0.032**
Group (*AMY*=0, *VOL*=1)	0.34	0.18	0.002	0.68	3.61	0.057
Daytime (early morning vs. late morning)[e]	-0.22	0.19	-0.58	0.18		
Daytime (early morning vs. early afternoon)[e]	-0.21	0.18	-0.55	0.15	4.47	0.21
Daytime (early morning vs. late afternoon)[e]	0.11	0.20	-0.30	0.51		

[a] Reference level is forest.
[b] z-transformed to mean = 0 and SD = 1 prior to model fitting; original mean (SD) was 0.42 (0.28).
[c] Reference level is adult male.
[d] z-transformed to mean = 0 and SD = 1 prior to model fitting; original mean (SD) was 0.51 (0.35).
[e] Reference level is early morning.
[f] z-transformed to mean = 0 and SD = 1 prior to model fitting; original mean (SD) was 0.47 (0.25).
[g] z-transformed to mean = 0 and SD = 1 prior to model fitting; original mean (SD) was 0.51 (0.34).

Table S2. Results of the GLMM exploring the effect of the habitat on macaques' social partner diversity (model 5). Shown are model estimates, standard errors (SE), lower and upper confidence intervals (CI) and test results of individual effects. Control variables are not interpreted. Significant p-values are shown in bold.

Predictor variable	Estimate	SE	lower CI	upper CI	X²	P
Intercept	-4.12	0.17	-4.51	-3.81		
Habitat (forest=0, plantation edge=1)	1.04	0.15	0.71	1.31	31.07	**< 0.001**
Control variables						
Feeding rate[a]	-1.08	0.07	-1.22	-0.94	115.11	**< 0.001**
Age-sex class (adult ♂ vs. adult ♀)[b]	0.70	0.15	0.41	1.00		
Age-sex class (adult ♂ vs. immature ♂)[b]	1.06	0.18	0.70	1.44	33.11	**< 0.001**
Age-sex class (adult ♂ vs. immature ♀)[b]	0.94	0.17	0.61	1.29		
Rank[c]	0.006	0.05	-0.10	0.11	0.01	0.90
Group (*AMY*=0, *VOL*=1)	-0.47	0.13	-0.75	-0.22	12.38	**< 0.001**
Daytime (early morning vs. late morning)[d]	0.03	0.11	-0.19	0.24		
Daytime (early morning vs. early afternoon)[d]	0.09	0.12	-0.17	0.33	6.94	0.074
Daytime (early morning vs. late afternoon)[d]	0.30	0.12	0.03	0.53		

[a] z-transformed to mean = 0 and SD = 1 prior to model fitting; original mean (SD) was 0.42 (0.28).
[b] Reference level is adult male.
[c] z-transformed to mean = 0 and SD = 1 prior to model fitting; original mean (SD) was 0.51 (0.35).
[d] Reference level is early morning.

Table S3. Results of the GLMM exploring the effect of habitat and its interaction with dominance rank and age-sex class on the macaques' individual scores of eigenvector centrality (model 6). Shown are model estimates, standard errors (SE), lower and upper confidence intervals (CI) as well as original and permuted p-values of the three-way interaction. Permuted p-values were obtained by comparing the observed regression coefficients with a distribution of 1,000 coefficients generated by randomly swapping the nodes of the social network prior to extracting centrality scores. Significance is indicated in bold.

Predictor variable	Estimate	SE	lower CI	upper CI	P	$P_{permuted}$
Intercept	0.34	0.07	0.20	0.48		
Predictors included in interaction						
Habitat (forest = 0, plantation edge = 1)	-0.26	0.10	-0.46	-0.07	[d]	[d]
Rank[a]	0.04	0.07	-0.11	0.19	[d]	[d]
Age-sex class (adult ♂ vs. adult ♀))[b]	0.21	0.09	0.03	0.40	[d]	[d]
Age-sex class (adult ♂ vs. immature ♀)[b]	0.14	0.10	-0.05	0.35	[d]	[d]
Age-sex class (adult ♂ vs. immature ♂)[b]	0.10	0.11	-0.12	0.30	[d]	[d]
Two-way interaction						
Habitat[c] * rank	-0.02	0.10	-0.22	0.19	[d]	[d]
Habitat[c] * age-sex class (adult ♂ vs. adult ♀)[b]	-0.08	0.12	-0.32	0.19	[d]	[d]
Habitat[c] * age-sex class (adult ♂ vs. immature ♀)[b]	0.05	0.14	-0.20	0.33	[d]	[d]
Habitat[c] * age-sex class (adult ♂ vs. immature ♂)[b]	0.21	0.15	-0.08	0.51	[d]	[d]
Rank * age-sex class (adult ♂ vs. adult ♀)[b]	-0.17	0.09	-0.36	0.01	[d]	[d]
Rank * age-sex class (adult ♂ vs. immature ♀)[b]	-0.19	0.10	-0.38	0.009	[d]	[d]
Rank * age-sex class (adult ♂ vs. immature ♂)[b]	-0.11	0.10	-0.31	0.09	[d]	[d]
Three-way interaction[e]						
Habitat[c] * rank * age-sex class (adult ♂ vs. adult ♀)[b]	0.27	0.13	0.02	0.53	0.038	**0.050**
Habitat[c] * rank * age-sex class (adult ♂ vs. immature ♀)[b]	0.43	0.14	0.16	0.72	0.002	**0.007**
Habitat[c] * rank * age-sex class (adult ♂ vs. immature ♂)[b]	0.26	0.14	-0.04	0.57	0.073	0.108

[a] z-transformed to mean = 0 and SD = 1 prior to model fitting; original mean (SD) was 0.50 (0.33).
[b] Reference level is adult male.
[c] Reference level is forest.
[d] Values are not shown because of having a very limited interpretation as they are part of the interaction.
[e] The global testing (LRT habitat * rank * age-sex class) revealed $X^2 = 11.50$, df = 3, P = 0.009.

Table S4. Results of the GLMMs exploring the effect of the habitat on three measures describing the mother-infant relationship in macaques, i.e. the proportion of contact time (model 7), the rate of mothers breaking contact (model 8) and the rate of mothers increasing distance (model 9). For all predictor variables except non-significant interactions, model estimates, standard errors (SE), lower and upper confidence intervals (CI) and test results of individual fixed effects are shown after removal of the non-significant interaction terms. Control variables are not interpreted. Significant p-values are shown in bold.

**Predictor variable**	**Estimate**	**SE**	**lower CI**	**upper CI**	**X^2**	**P**
Contact time (model 7)						
Intercept	0.85	0.32	0.25	1.49		
Habitat (forest vs. plantation edge)[a]	1.98	0.31	1.40	2.56	47.69	**< 0.001**
Habitat (forest vs. plantation interior)[a]	3.74	0.19	3.40	4.15		
Control variables						
Rate of socializing[b]	-0.12	0.19	-0.47	0.25	0.37	0.54
Infant age[c]	-2.18	0.14	-2.46	-1.92	31.93	**< 0.001**
Infant sex (♂ = 0, ♀ = 1)	0.44	0.44	-0.36	1.32	1.01	0.32
Parity (multiparous = 0, primiparous = 1)	-1.31	0.45	-2.22	-0.44	5.61	**0.018**
Mother's rank[d]	0.25	0.20	-0.13	0.66	1.38	0.24
Daytime (early morning vs. late morning)[e]	-0.76	0.36	-1.41	0.08		
Daytime (early morning vs. early afternoon)[e]	-0.06	0.38	-0.83	0.71	4.56	0.21
Daytime (early morning vs. late afternoon)[e]	0.06	0.31	-0.53	0.69		
Interaction excluded due to non-significance						
Habitat (forest vs. plantation edge)[a] * infant age	0.12	0.38	-0.65	0.93	0.10	0.95
Habitat (forest vs. plantation interior)[a] * infant age	-0.02	0.35	-0.75	0.75		
Mother breaks contact (model 8)						
Intercept	0.10	0.23	-0.39	0.50		
Control variables						
Rate of socializing[b]	-0.11	0.07	-0.28	0.02	2.52	0.11
Infant age^2	-0.35	0.09	-0.56	-0.19	15.03	**< 0.001**
Infant sex (♂ = 0, ♀= 1)	-0.05	0.19	-0.42	0.33	0.07	0.79
Parity (multiparous = 0, primiparous = 1)	0.72	0.18	0.38	1.08	14.00	**< 0.001**
Mother's rank[d]	-0.26	0.09	-0.47	-0.08	8.21	**0.004**
Daytime (early morning vs. late morning)[e]	0.31	0.29	-0.29	0.87		
Daytime (early morning vs. early afternoon)[e]	0.41	0.31	-0.18	1.10	5.26	0.15
Daytime (early morning vs. late afternoon)[e]	0.45	0.20	0.05	0.83		
Predictors included in interaction						
Habitat (forest vs. plantation edge)[a]	-0.66	0.23	-1.14	-0.28	[f]	[f]
Habitat (forest vs. plantation interior)[a]	-1.04	0.29	-1.66	-0.53	[f]	[f]
Infant age[c]	-0.001	0.14	-0.28	0.27	[f]	[f]
Two-way interaction						
Habitat (forest vs. plantation edge)[a] * infant age	0.82	0.24	0.36	1.33	17.22	**< 0.001**
Habitat (forest vs. plantation interior)[a] * infant age	0.76	0.21	0.33	1.21		
Interaction excluded due to non-significance						
Habitat (forest vs. plantation edge)[a] * infant age^2	-0.08	0.21	-0.59	0.31	0.14	0.93
Habitat (forest vs. plantation interior)[a] * infant age^2	-0.04	0.25	-0.62	0.38		
Mother increases distance (model 9)						
Intercept	1.52	0.28	0.94	2.03		
Control variables						
Rate of socializing[b]	-0.18	0.06	-0.31	-0.07	8.04	**0.005**
Infant age^2	-0.51	0.10	-0.71	-0.34	16.00	**< 0.001**
Infant sex (♂ = 0, ♀ = 1)	-0.48	0.44	-1.34	0.40	1.24	0.27
Parity (multiparous = 0, primiparous = 1)	0.72	0.43	-0.18	1.58	2.37	0.12
Mother's rank[d]	-0.18	0.20	-0.57	0.19	0.72	0.40
Daytime (early morning vs. late morning)[e]	-0.16	0.21	-0.57	0.23		
Daytime (early morning vs. early afternoon)[e]	-0.29	0.18	-0.65	0.08	3.88	0.28
Daytime (early morning vs. late afternoon)[e]	0.07	0.16	-0.25	0.41		
Predictors included in interaction						
Habitat (forest vs. plantation edge)[a]	-1.80	0.33	-2.55	-1.14	[f]	[f]
Habitat (forest vs. plantation interior)[a]	-2.35	0.47	-3.30	-1.54	[f]	[f]
Infant age[c]	0.79	0.17	0.49	1.11	[f]	[f]
Two-way interaction						
Habitat (forest vs. plantation edge)[a] * infant age	1.04	0.20	0.62	1.48	26.21	**< 0.001**
Habitat (forest vs. plantation interior)[a] * infant age	1.21	0.21	0.72	1.74		
Interaction excluded due to non-significance						
Habitat (forest vs. plantation edge)[a] * infant age^2	0.10	0.20	-0.44	0.47	0.29	0.86
Habitat (forest vs. plantation interior)[a] * infant age^2	-0.06	0.26	-0.78	0.46		

[a] Reference level is forest.
[b] z-transformed to mean = 0 and SD = 1 prior to model fitting; original mean (SD) was 0.07 (0.17).
[c] z-transformed to mean = 0 and SD = 1 prior to model fitting; original mean (SD) was 80.98 (49.42).
[d] z-transformed to mean = 0 and SD = 1 prior to model fitting; original mean (SD) was 0.45 (0.32).
[e] Reference level is early morning.
[f] Values are not shown because of having a very limited interpretation as they are part of the interaction.
